# Process-specific mechanisms of vertically oriented graphene growth in plasmas

**DOI:** 10.3762/bjnano.8.166

**Published:** 2017-08-10

**Authors:** Subrata Ghosh, Shyamal R Polaki, Niranjan Kumar, Sankarakumar Amirthapandian, Mohamed Kamruddin, Kostya (Ken) Ostrikov

**Affiliations:** 1 Surface and Nanoscience Division, Materials Science Group, Indira Gandhi Centre for Atomic Research - Homi Bhabha National Institute, Kalpakkam - 603102, India; 2 Materials Physics Division, Materials Science Group, Indira Gandhi Centre for Atomic Research - Homi Bhabha National Institute, Kalpakkam - 603102, India; 3 School of Chemistry, Physics and Mechanical Engineering, Queensland University of Technology, Brisbane QLD 4000, Australia; 4 CSIRO-QUT Joint Sustainable Processes and Devices Laboratory, Lindfield NSW 2070, Australia

**Keywords:** activation energy, plasmas, residual stress, vertical graphene nanosheets, wettability

## Abstract

Applications of plasma-produced vertically oriented graphene nanosheets (VGNs) rely on their unique structure and morphology, which can be tuned by the process parameters to understand the growth mechanism. Here, we report on the effect of the key process parameters such as deposition temperature, discharge power and distance from plasma source to substrate on the catalyst-free growth of VGNs in microwave plasmas. A direct evidence for the initiation of vertical growth through nanoscale graphitic islands is obtained from the temperature-dependent growth rates where the activation energy is found to be as low as 0.57 eV. It is shown that the growth rate and the structural quality of the films could be enhanced by (a) increasing the substrate temperature, (b) decreasing the distance between the microwave plasma source and the substrate, and (c) increasing the discharge power. The correlation between the wetting characteristics, morphology and structural quality is established. It is also demonstrated that morphology, crystallinity, wettability and sheet resistance of the VGNs can be varied while maintaining the same sp^3^ content in the film. The effects of the substrate temperature and the electric field in vertical alignment of the graphene sheets are reported. These findings help to develop and optimize the process conditions to produce VGNs tailored for applications including sensing, field emission, catalysis and energy storage.

## Introduction

Vertical graphene nanosheets (VGNs) consist of interconnected 3D porous networks of vertically oriented graphitic sheets, which are aligned perpendicularly to the substrates, containing 3–12 graphene layers [1]. The dimensions of the nanosheets are typically up to a few micrometers. The interconnected vertical 3D network is anchored onto a nanometer-thick graphitic base layer grown on the VGNs–substrate interface. Their unique properties such as large surface area, non-agglomerated structure, sharp edges, excellent thermal and electrical properties, thermal and electrochemical stability and ease in functionalization make VGNs promising candidates for a wide range of applications that include field emission, sensing, energy storage, metamaterials, biomedical and other devices [1–7].

Efficient utilization of VGNs depends on their effective surface area, which is determined by two major factors: (a) vertical sheet density and (b) intersheet spacing. Enhancing these two factors improves the electron transfer kinetics and hence electrochemical properties [2,8]. Moreover, electrical, structural and optical properties of VGNs can be tuned by controlling the vertical sheet density, structural imperfections and the chemical nature of edge states [9–11]. Shih et al. [12] have reported the importance of optimized growth for better field-emission properties of VGNs. The culturing rate of cancer cervical cells was found to strongly depend on the density of VGNs by Watanabe and co-workers [13]. Recently, Bo et al. [14] reported the tunability of wetting properties from hydrophobic to hydrophilic by reducing the intersheet spacing of VGNs for enhanced water purification and supercapacitive performance. Hence, the aim of research on VGNs is to achieve a pre-determined structure for suitable applications, which depends on the hierarchical organization of nanostructures. Such self-organization can be realized by controlling the process parameters during growth.

Plasma-enhanced chemical vapor deposition (PECVD) is one of the most suitable techniques for the transfer-free and catalyst-free growth of VGNs at low temperature [15–20]. Various research groups reported the growth mechanism of VGNs during PECVD [21–24]. In brief, the growth is initiated with a buffer layer consisting of amorphous carbon and carbon onion structures, nanographitic (NG) island formation, or through carbide formation. The factors responsible for the vertical growth are stress relaxation through NG islands, inherent electric field and thermophoretic force along with supersaturation of the carbon source and a simultaneous etching process by nascent hydrogen [21–25].

Based on the experimental observations, a phenomenological four-stage model was proposed [24]. In the plasma-assisted growth of carbon nanostructures, the hydrocarbon precursor dissociates under plasma and forms reactive radicals/ions. Transport mechanism of these plasma species and growth kinetics of carbon nanomaterials in PECVD has been extensively explained by Munoz and co-workers [26]. The density and energy of these plasma species depend on the plasma power, position of the substrate in the plasma plume and feedstock gas composition. The substrate defines the surface reaction kinetics, thus the nature of the substrate and its temperature play major role in the growth [24]. Therefore, these key factors affect the final morphology and structural quality.

Several research groups have observed the effect of various process parameters such as carrier gas, nature of substrate, total pressure and microwave power on the growth of VGNs. However, the plasma chemistry and chemical reactions with the substrate surface during growth are still a matter of study [9,27–50]. The role of gas composition, deposition time and nature of substrate on the growth of VGNs has been discussed [24,27,47]. The first two parameters underpin the two competing disorder-related mechanisms. These mechanisms contribute to the defect band intensity through graphite structure amorphization and growth orientation [27,47].

The substrate properties such as surface energy, thermal conductivity and atomic density play a major role in determining the structure and morphology of substrate-supported VGNs [24]. In general, the substrate temperature defines the energy and mobility of the plasma gases/species. It has been reported that the vertical structure cannot be grown from gaseous precursors at substrate temperatures lower than 630 °C [51]. Cho et al. [9] have shown that a variation of the plasma discharge power and operating pressure lead to tunable number density of vertical sheets determining the electrical properties, while the height was maintained constant. The intersheet spacing and surface area of VGNs can be controlled by the plasma power, while excess power leads to amorphization due to bombardment of more energetic ions and chemical etching [52]. However, the mechanisms of the growth and the formation of the final chemical structure and morphology of the VGNs were not explained. Therefore, the scope of the present investigation is to optimize the plasma process parameters and relate them with the morphology and structure of the VGNs, in particular using the plasma chemistry considerations.

Here we aim to study the role of the three key parameters such as the substrate temperature, microwave power and the distance from plasma source to substrate in electron cyclotron resonance (ECR)-PECVD to control the morphology and structure of VGNs. In this study, the evolution and mechanism of catalyst-free growth of VGNs on the nanographitic structure is described. The systematic characterization of the morphology and structure was carried out by field-emission scanning electron microscopy (FESEM), high-resolution transmission electron microscopy (HRTEM) and Raman spectroscopy. Contact angle and electrical resistance measurements of the VGNs are carried out as well.

## Results and Discussion

### Growth and optimization

#### Case I: Influence of growth temperature

**Figure 1:**
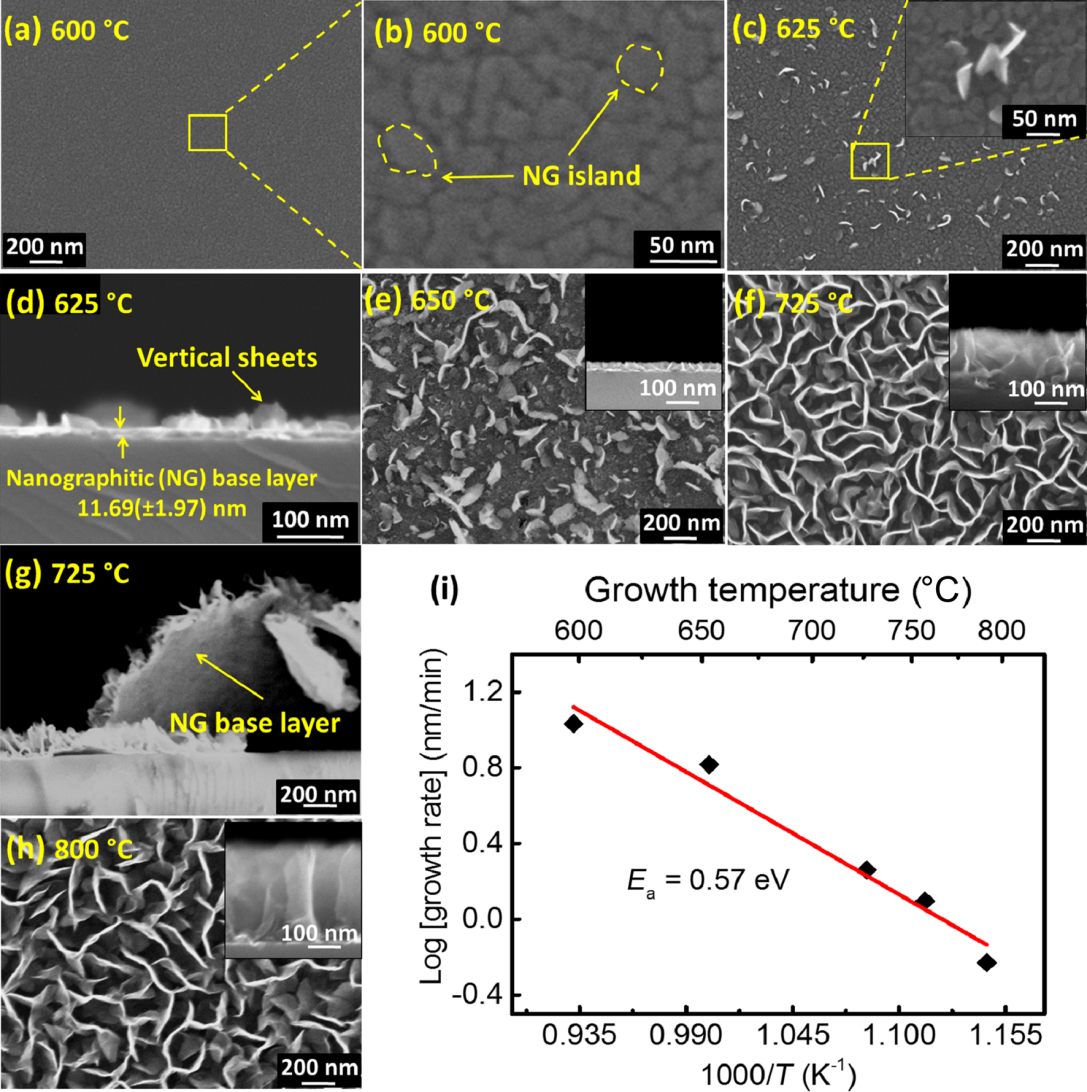
Scanning electron micrographs of the samples grown at different substrate temperatures (a,b) 600 °C, (c) 625 °C, (d) 625 °C (cross section), (e) 650 °C, (f) 725 °C, (g) 725 °C (cross section) and (h) 800 °C, while deposition time, microwave power and distance from plasma source to substrate were 30 min, 320 W and 30 cm, respectively. (i) Arrhenius plot of the growth rate versus the inverse of the substrate temperature.

We investigated the early-stage nucleation and growth of VGNs over a substrate temperature range of 600–800 °C under CH_4_/Ar gas environment for 30 min, while the plasma power and distance from the substrate were maintained at 320 W and 30 cm, respectively. Figure 1a–h shows the FESEM images of the films grown at various substrate temperatures. It is evident that an increase in the substrate temperature from 600 to 800 °C leads to a transformation of the structures from continuous nanographitic (NG) layers to interconnected network of vertical graphene. NG islands are clearly seen in images taken at higher magnifications (Figure 1b). The thickness of the NG film is measured to be 17 ± 2 nm. The average size and density of NG island are observed to be around 28 ± 18 nm and 925 ± 161 μm^−2^, respectively. The island densities are calculated by averaging out the number of islands in a 0.2 μm × 0.2 μm area at several places of the micrograph using the ImageJ software. In this study, NG structures were not observed below 600 °C and this is explained by adverse etching of graphene by hydrogen radicals in the plasma, which dominates over the graphene growth at lower temperatures [46]. Figure 1c shows the vertical sheets nucleated from the grain boundaries. This is attributed to a stress-release mechanism of the coalescing graphene/NG islands [22,24]. In addition, the inherent localized electric field due to the plasma and the associated polarization and thermophoretic effects drive the growth of the graphene sheets in the normal direction to the substrate [21,51]. As shown in Figure 1d, vertical sheets of a height of 37 ± 9 nm form on a NG layer. Here the NG base-layer thickness of 11 ± 2 nm is smaller compared to the one grown at 600 °C (17 ± 2 nm). Hence, it is inferred that the thickness of the NG layer decreases with the growth temperature. In addition, the average sizes of the islands on the NG layer are measured to be around 40 ± 18 nm with a density of 768 ± 68 μm^−2^. Further increase in temperature to 650 °C (Figure 1e) led to a larger number of vertical sheets as compared to the previous sample grown at 625 °C. Sheet-like structures formed with higher growth rates at 725 °C, as shown in Figure 1f.

Direct evidence for the NG base layer below the vertical sheets is shown in Figure 1g. Moreover, the sheets started to interlace resulting in an increase inter-sheet spacing while maintaining the sheet-like features at 800 °C (Figure 1h). In order to quantify the vertical growth, the growth rate is plotted as a function of the temperature in Figure 1i. This dependence can be described by the Arrhenius equation. The activation energy is calculated from this plot. The activation energy for the vertical growth is found to be 0.57 eV. Faster nucleation and growth of VGNs were reported on carbon-based substrates compared to non-carbon substrates [48]. To substantiate this, we carried out growth of VGNs on a SiC substrate and found that the height of VGNs on SiC is 270 nm after 30 min of growth. The height of VGNs on SiO_2_ is found to be 216 nm. Therefore, the low activation energy of the vertical growth is due to the fact that vertical growth started from a continuous NG layer rather than directly from the substrate surface.

**Figure 2:**
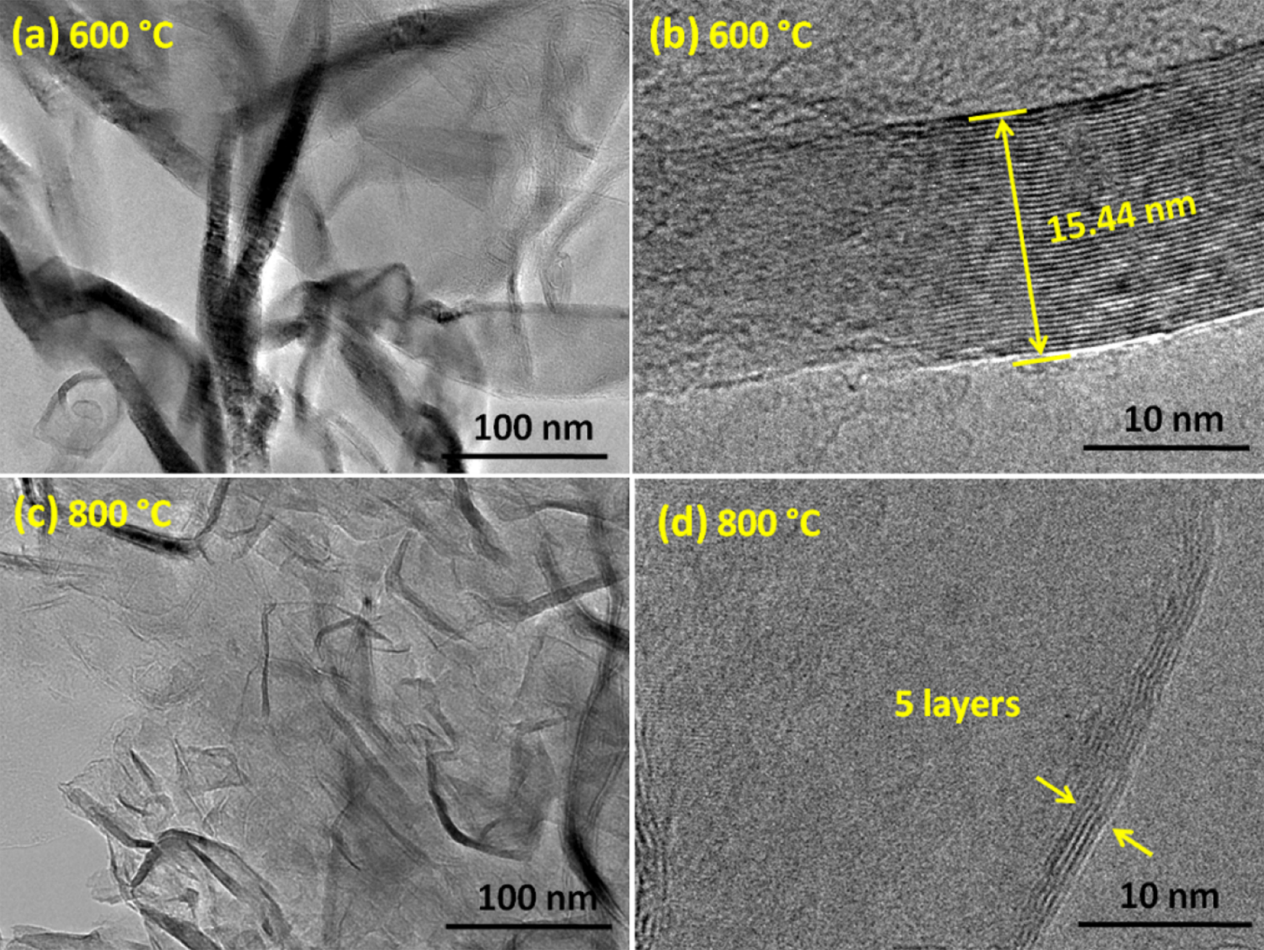
Transmission electron micrograph of (a,b) the nanographitic layer and (c,d) vertical graphene nanosheets (VGNs). Panel (b) shows the thickness of the nanographitic layer and panel (d) shows that the top edges of VGNs consist of only five graphene layers.

Figure 2a,b and Figure 2c,d show transmission electron micrographs (TEM) of the films grown at 600 and 800 °C, respectively. The HRTEM in Figure 2b clearly shows the thickness of a NG layer grown at 600 °C to be 15.44 nm, matching well with that obtained from the SEM cross sections (17 ± 2 nm). TEM of VGNs grown at 800 °C, shown in Figure 2c, reveals transparent sheets with the corrugated and wrinkled structure. The HRTEM in Figure 2d clearly evidences the presence of 2–5 graphene layers at the top edges.

**Figure 3:**
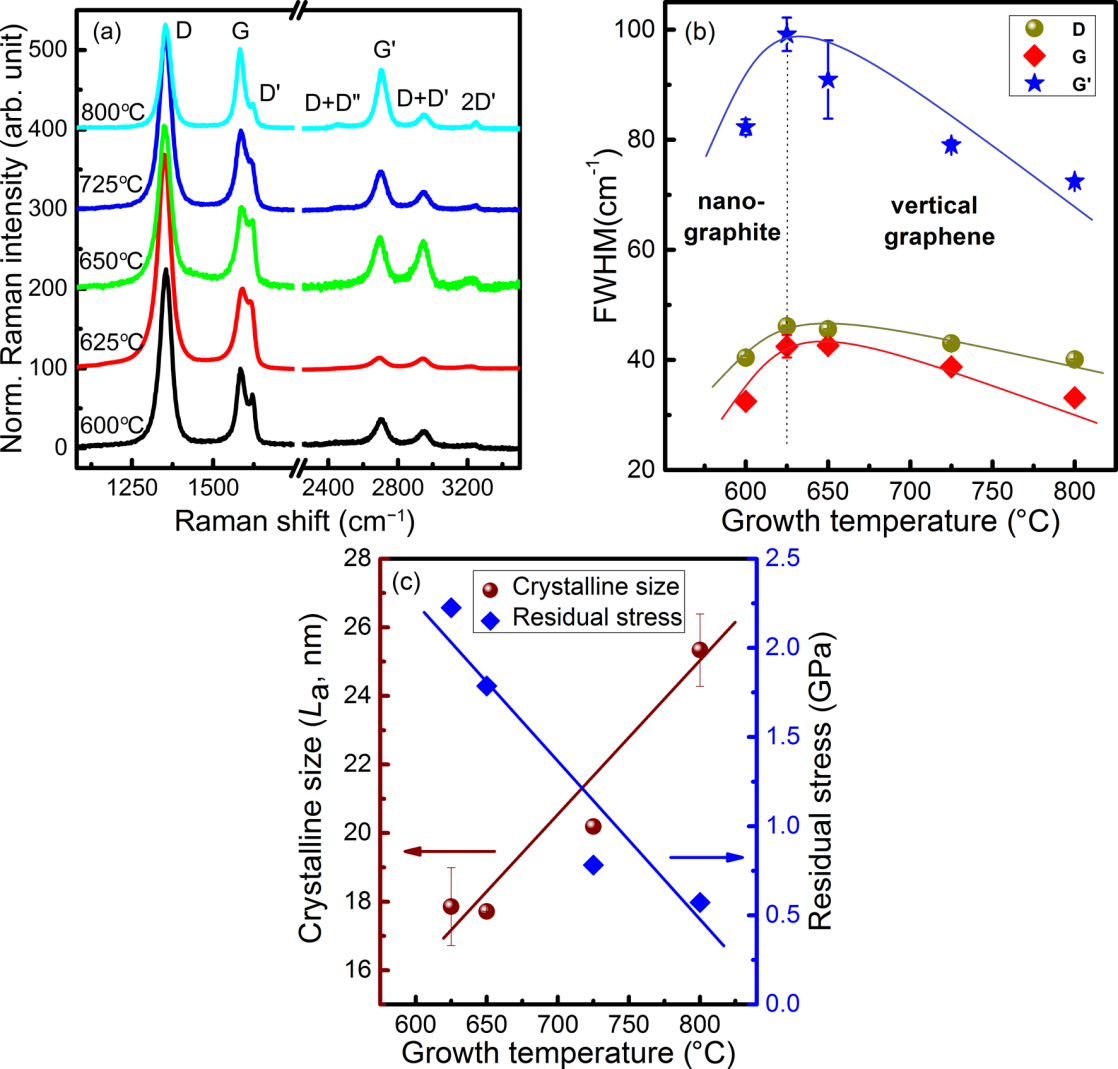
(a) Raman spectra of VGNs grown at different substrate temperatures. (b) Values of full widths at half maximum (FWHM) of D, G, and G′ bands, and (c) residual stress and crystallite size as a function of the growth temperature. Solid lines are to guide the eye.

Figure 3 presents the Raman spectra of the samples grown at various temperatures. The first-order Raman spectra of VGNs feature a D peak at 1350 cm^−1^, a G peak at 1580 cm^−1^ and a D′ peak at 1620 cm^−1^. The second-order Raman spectra exhibit D + D″ (ca. 2450 cm^−1^), G′ (ca. 2705 cm^−1^), D + D′ (ca. 2948 cm^−1^) and 2D′ (ca. 3244 cm^−1^) peaks. The defect-related peaks (D, D′, D″ and the overtones) are attributed to high edge density, structural defects and disorder such as vacancies and strained hexagonal/non-hexagonal (pentagon or heptagon) distortions. These factors lead to the non-uniformity, corrugation and twisting as shown in SEM and TEM images (Figure 1 and Figure 2) [24,43]. The presence of G bands and G′ bands confirms the graphitic nature in the film. It is observed that the D′-band intensity increases up to 625 °C and then decreases at higher growth temperatures (Figure 3a). The full width at half maximum (FWHM) values of D, G and G′ band follow a similar trend (Figure 3b). The in-plane crystallite size, *L*_a_, is estimated using Equation 1 [53]:

[1]
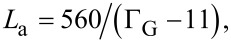


where Γ_G_ is the FWHM of the G band. *L*_a_ is found to 26.1 nm for the film grown at 600 °C, which agrees with the SEM observation (grain size = 28 nm). The in-plane crystallite size increases with the growth temperature, as shown in Figure 3c. At 625 °C the vertical sheets started to form, accompanied by an increase of defect density due to dominance of edges. This can be described by the formation of low-dimensional, extremely small nanosheets where strain might be high due to the generation of intrinsic defects as discussed below in this paper. However, better graphitization is noticed at high temperatures when the number of edge-related defects is reduced.

The residual stress for the vertical growth is calculated using the empirical Equation 2 [54]:

[2]
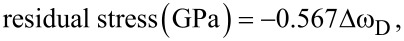


where Δω_D_ is the displacement of the D band in the Raman spectra. Here, the position of D band (ω_D_) of VGNs grown at 600 °C is considered as a reference point. It is found from Figure 3c that the residual stress decreases with increasing growth temperature. The residual stress is described as internal stress in nanostructured materials.

The generation of residual stress during growth can be attributed to: (i) thermal stress, which occurs because of the difference in thermal expansion coefficient between the substrate and the VGNs; (ii) ion-bombardment generated defects; and (iii) lattice mismatch between the substrate and the nanographitic layer [54]. More importantly, it is noticed that the crystallite size is inversely proportional to the intrinsic residual stress. Stress decreases with the improvement in crystallinity to facilitate grain growth in nanocrystalline materials. This trend, clearly shown in Figure 3c, implies that the initial growth of the vertical sheets can be attributed to the relaxation of stress that starts at grain boundaries of NG islands. The higher growth rates at high temperature could be due to chemisorption and diffusion of carbon atoms to the edge of vertical sheets rather than the upward push by the released stress. The edges are partially hydrogen-terminated or closed by folded atomic carbon layers [23,55]. Edges of these sheets are reactive due to structural defects, dangling bonds and open co-valency of carbon atoms, which affects the diffusion and adsorption of carbon atoms. Further, growth takes place by the material supplied through diffusion-assisted adsorption of carbon atoms at the edges of the nanosheets. Moreover, the sticking coefficient of the carbon adatoms controls the growth rate [23]. These factors promote the extended growth of graphene-like structures. Finally, the closure of these open edges (seamless ends of two adjacent monolayers) curtails the growth and, in turn, determines the height of the sheets [23].

The fraction of sp^3^ bonds present in the samples is calculated from the empirical Equation 3 [54]:

[3]
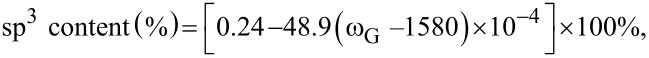


where ω_G_ is the position of the G peak. The sp^3^ content of the samples is found to be in the range of 19 to 22%, and is considered to be localized at “in-wall-boundary” and “in-grain-boundary” regions [56]. Therefore, it is possible to tune the morphology from planar to vertical networks by altering the growth temperature while maintaining the same sp^3^ content.

**Figure 4:**
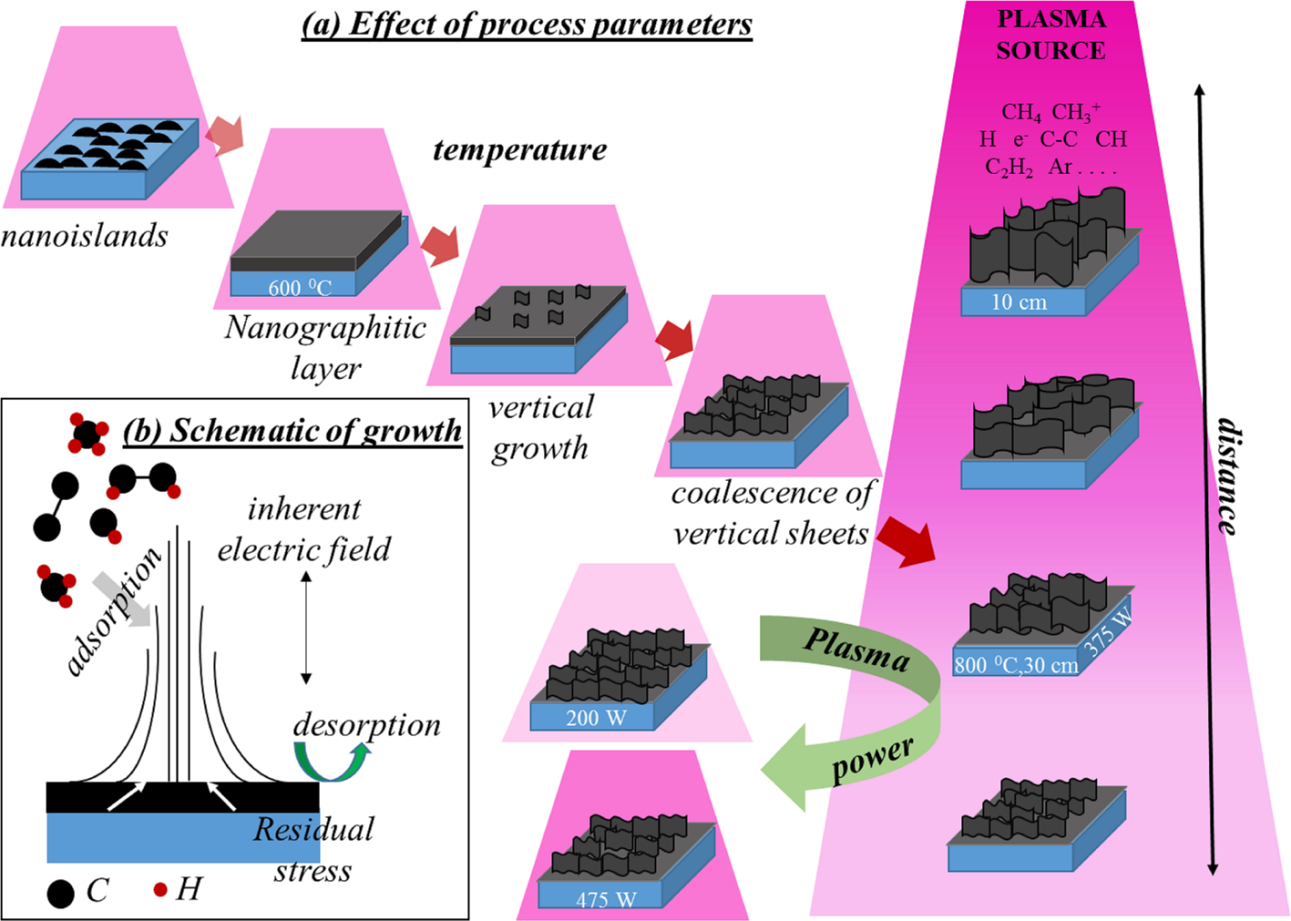
Schematic of (a) effect of process parameters on the growth and (b) growth mechanism of vertical graphene nanosheets.

According to the above results, the growth mechanism of VGNs, shows as schematic in Figure 4, can be explained as follows: The ECR-CVD creates highly energetic plasma species (e.g., C*_x_*H*_y_*, C_2_, CH, CH^+^, H, H^+^) through electron-impact dissociation of CH_4_ [26]. Electrons move faster than the ions, hence the surface acquires a distributed negative electric charge [57]. This negatively charged surface produces microscopic electric fields that accelerate the ions to achieve higher energies. Therefore, the electron gas acts as an effective negative bias field to generate high-energy ions. The electrons and ions interact with other plasma species through chemical reactions that lead to molecular decomposition and transformative re-assembly [57]. The species that influence the growth of carbon nanostructures in plasmas are C_2_ and CH, as well as atomic and molecular hydrogen [26]. The rapid nucleation of nanoislands, self-organization and coalescence between them take place through direct adsorption and surface diffusion of carbon-containing species on the substrate surface [24]. Hence, the commonly observed (e.g., through electron microscopy) reduction in thickness of the NG layer is related to the change in adsorption–desorption balance and the longer surface residence time for the species at lower temperatures [58]. Higher growth temperatures promote the migration of the plasma-generated species reaching the substrate and increase the probability of chemisorption, which is favorable for the formation of stable nanostructures in interconnected vertical networks normal to the substrate. However, the heating effect due to plasma is not considered here as the plasma power and deposition distance are maintained constant. Hence, this result is related to the increased mobility of surface atoms such that the rates of adsorption and surface chemical reactions become higher at high temperatures; this reduces the surface energy of the substrate. Similar transformations of islands to VGNs have also been reported previously in the studies of time-dependent growth of VGNs [27].

#### Case II: Influence of deposition distance

**Figure 5:**
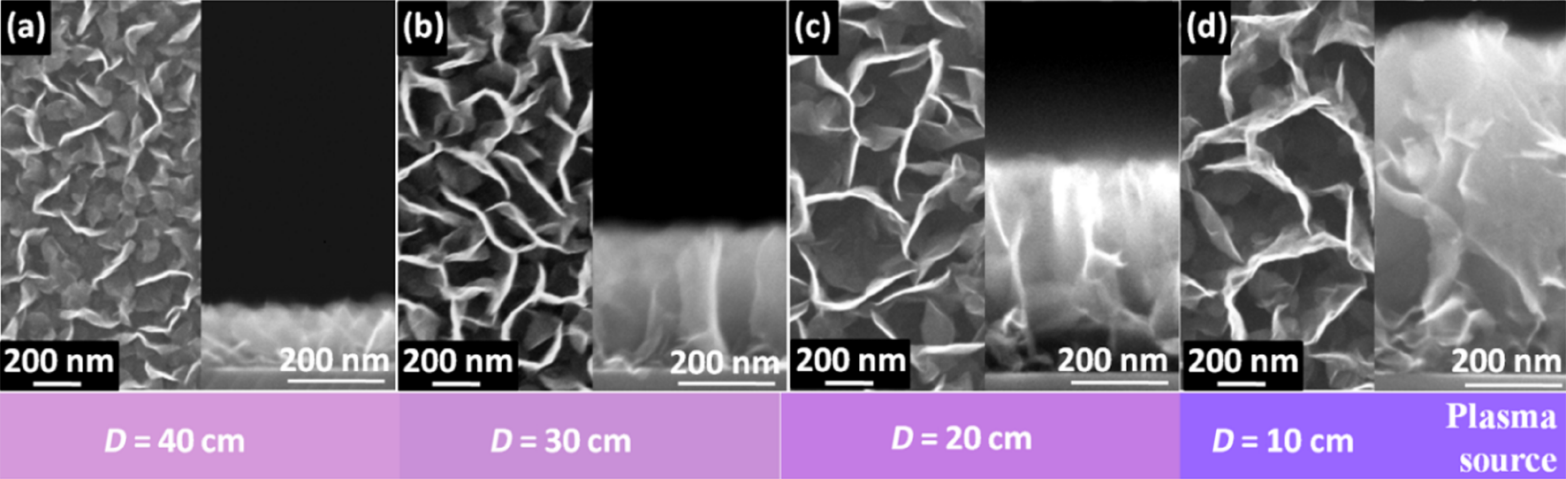
Scanning electron micrographs and corresponding cross sections of VGNs grown at different distances from the plasma source, namely, (a) 40 cm, (b) 30 cm, (c) 20 cm, and (d) 10 cm.

Figure 5 depicts the morphology of VGNs deposited at various distances between the plasma source and the substrate. It is observed that the number density of vertical sheets decreases with the distance, while the dimensions of the sheets and intersheet spacing of VGNs increase. The intersheet spacing between the vertical sheets increases from 84 ± 26 nm to 336 ± 121 nm as the distance is reduced. In addition, a dramatic increase in growth rates is found and it increases from 5 to 25 nm/min as the distance is reduced from 40 to 10 cm.

**Figure 6:**
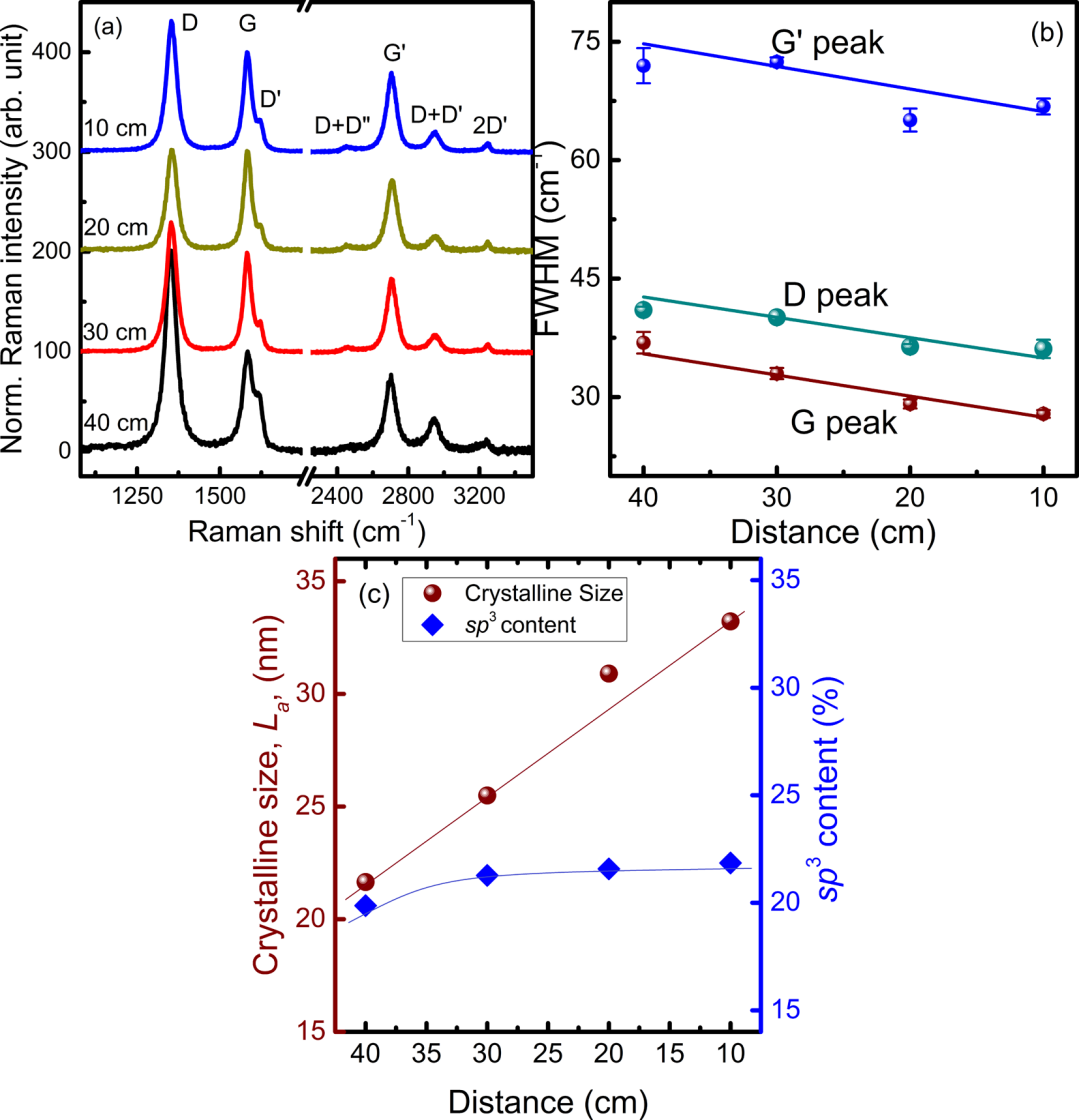
(a) Raman spectra of VGNs grown at different distance, (b) variation of FWHM of D, G, and G′ band and (c) crystallite size and sp^3^ content with respect to the distance from the plasma source to the substrate. Solid lines are to guide the eye.

The Raman spectra of VGNs grown at different distances are presented in Figure 6a. The FWHM of D, G and G′ peak decrease with distance, as shown in Figure 6b. The lowest FWHM of G peak is found to be 27.9 cm^−1^ for the sample grown at a distance of 10 cm. Larger crystallite sizes were obtained for the film grown at a closer distance, while the sp^3^ content remains almost equal, as shown in Figure 6c. This relationship clearly indicates the improved crystallinity of the samples produced at a lower distance from the plasma source.

The above experimental observations can be explained as follows: The inherent near-surface electric field is one of major driving factors for the growth of nanostructures under plasma conditions. Nanoparticles in plasma also experience a thermophoretic force due to the temperature gradients. Because the inherent electric field become stronger at higher energies and fluxes, positioning of the substrate is important since the plasma characteristics, such as the energy and density of the reactive species vary [59]. At higher distances, time for transporting carbon species (e.g., C_2_) from the plasma source to the substrates is high and the species may recombine. Under such conditions, radical species responsible for VGN growth may recombine and/or lose reactivity. Therefore, in low-density plasmas the carbon species can deposit into the spaces between the vertical graphene sheets.

However, at shorter distances the density of the plasma is high and plasma-generated carbon species attach to the reactive edges of the vertical sheets through chemisorption and diffusion, thus promoting the growth of crystalline sheets. In contrast, hydrogen species, mostly radicals, chemically etch the small flakes and amorphous carbon, which results in the increase in intersheet spacing between the VGNs [60]. Indeed, at shorter deposition distances, the substrate surface experiences a stronger electric field and larger amounts of carbon species can reach the surface without significant recombination. This in turn leads to the higher growth rate and larger crystallite sizes.

#### Case III: Effect of microwave power

**Figure 7:**
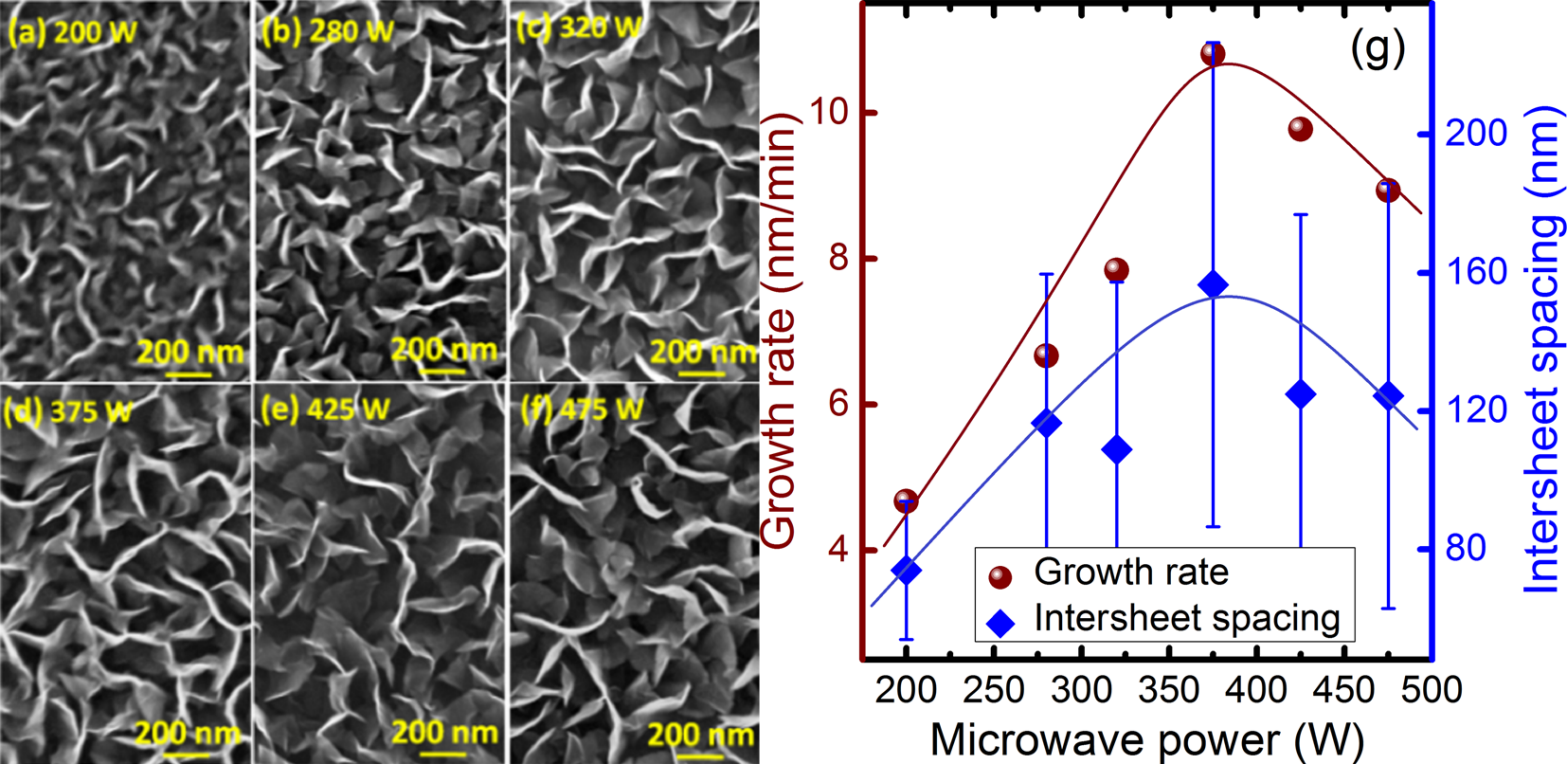
Scanning electron micrographs of VGNs grown at microwave powers of (a) 200 W, (b) 250 W, (c) 320 W, (d) 375 W, (e) 425 W and (f) 475 W; (g) variation of the growth rate and intersheet spacing as a function of the microwave power. Solid lines are to guide the eye.

Figure 7a–f shows the spatial distribution of vertical sheets as obtained by FESEM. Figure 7g is the plot of the growth rate and intersheet spacing as a function of the MW power. These observations suggest that the growth rates and areal density can be controlled by changing the MW power. Interestingly, higher growth rate and intersheet spacing are found in the sample grown at 375 W and both parameters decrease at higher powers [52].

**Figure 8:**
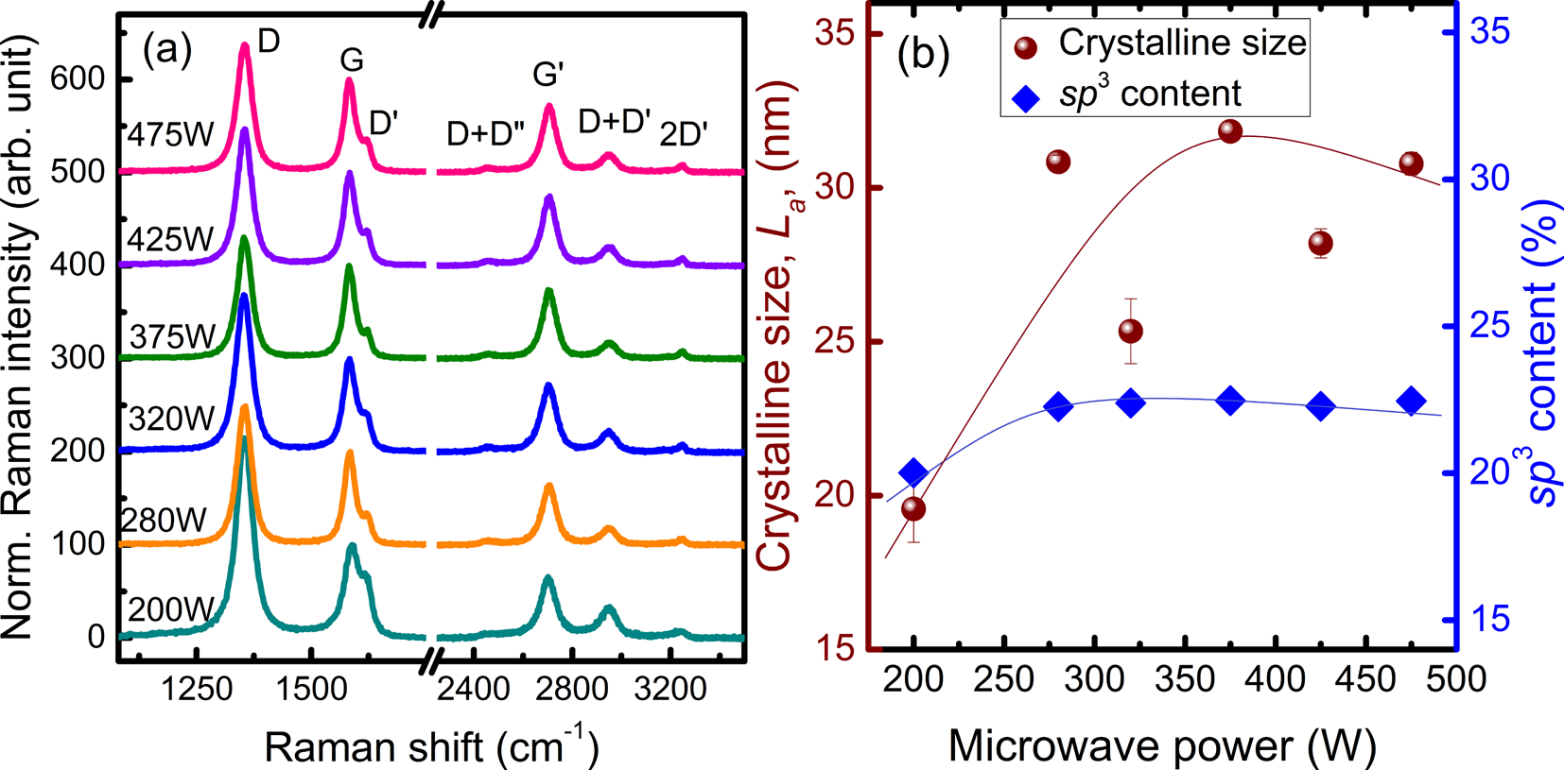
(a) Raman spectra of VGNs grown at a microwave power of 200 W, 250 W, 320 W, 375 W, 425 W and 475 W; (b) variation of crystallite size and sp^3^ content as a function of the microwave power. Solid lines are to guide the eye.

Figure 8a represents the Raman spectra of VGNs grown at different power. The results of Raman spectroscopy of these samples do not follow any consistent trends in FWHM, peak position and crystallite size. However, lower FWHM, larger crystallite size and least intense D′ peak are found for the sample grown at 375 W (Figure 8b). The sp^3^ content varies in the range of 20–22% for the samples grown at different MW power (Figure 8b).

The increase in plasma power enhances the decomposition rates, density, momentum and energy of ions, electrons and neutral species. The substrate temperature is kept constant at 800 °C for each MW power. Plasma exposure can also substantially increase the substrate temperature and electric field [29,34]. Apart from the substrate temperature, the microwave power can also increase the surface adatom mobility and more effectively accelerate electrons towards the substrate [59]. Indeed, the growth of VGNs is mainly based on the competition between deposition rate of carbon species, etching rate of carbon species by nascent H produced in the plasma and sputtering by highly energetic ion bombardment. Ion bombardment induced sputtering is negligible at lower ion energies and pronouncedly displaces C atoms from their stable position at higher ion energies [59]. The hydrogen species under plasma play a prominent role during the NG structure growth using PECVD by means of: (i) reducing the formation of undesirable form of carbon species, (ii) reducing the amount of C_2_ species by recombining with them to form CH*_x_* radicals, and (iii) etching of sp^2^-C, sp^3^-C and amorphous C at different rates. Hence, C/H ratios have a significant impact on morphology and graphitization, which enhance the quality of the structure.

The initial increase in growth rate and crystallite size with MW power is due to the increase in availability of C_2_ radicals and an optimal C/H ratio. At higher powers (above 375 W), the amount and energy of hydrogen species also increases, which reduces the density of C_2_ radicals. This eliminates the carbon species and, simultaneously, ion-induced sputtering takes place during the deposition [52]. These factors reduce the growth rate of VGNs and also modify the morphology and structure. Similar observations suggest that the planar graphitic structure transformed to VGNs and growth rate decreased above a certain plasma power [61]. Thus, the amount of hydrogen species has to be precisely controlled by tuning the MW power to achieve the optimum VGN structure with the desired morphology.

#### Wetting properties

**Figure 9:**
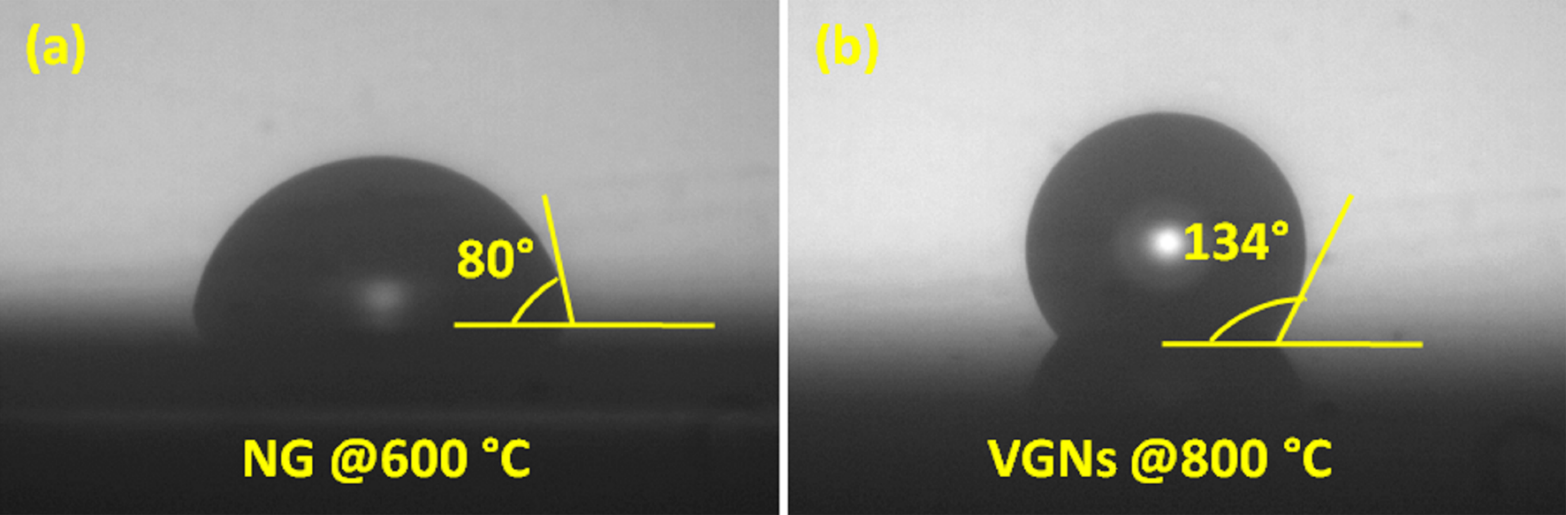
Water contact angle for the (a) nanographitic film (NG), grown at 600 °C and (b) VGNs, grown at 800 °C.

The desired wetting properties of materials are one of the important aspects from the application point of view. This wetting property depends on combined effect of several extrinsic and intrinsic factors including morphology, topography, surface texture, chemical structure and defects. Here, samples grown at 600 °C showed a (contact angle) CA of 80°, indicating hydrophilic behavior (Figure 9a). However, this value significantly increased to 134° for the sample grown at 800 °C, indicating near-superhydrophobic nature (Figure 9b). Such distinct characteristic might be explained by surface morphology, intersheet spacing, chemical structure, oxygen functionality and crystallinity [62]. The in-depth analysis of the CA behavior is outside the scope of this paper. The near-superhydrophobic behavior most likely originates from the effects of the improved crystallinity and increased intersheet spacing between VGNs. The contact angle is found to vary from 127° to 134° for the other studied samples. The improvement in hydrophobicity is significant and can be considered for many useful applications.

#### Electrical properties

**Figure 10:**
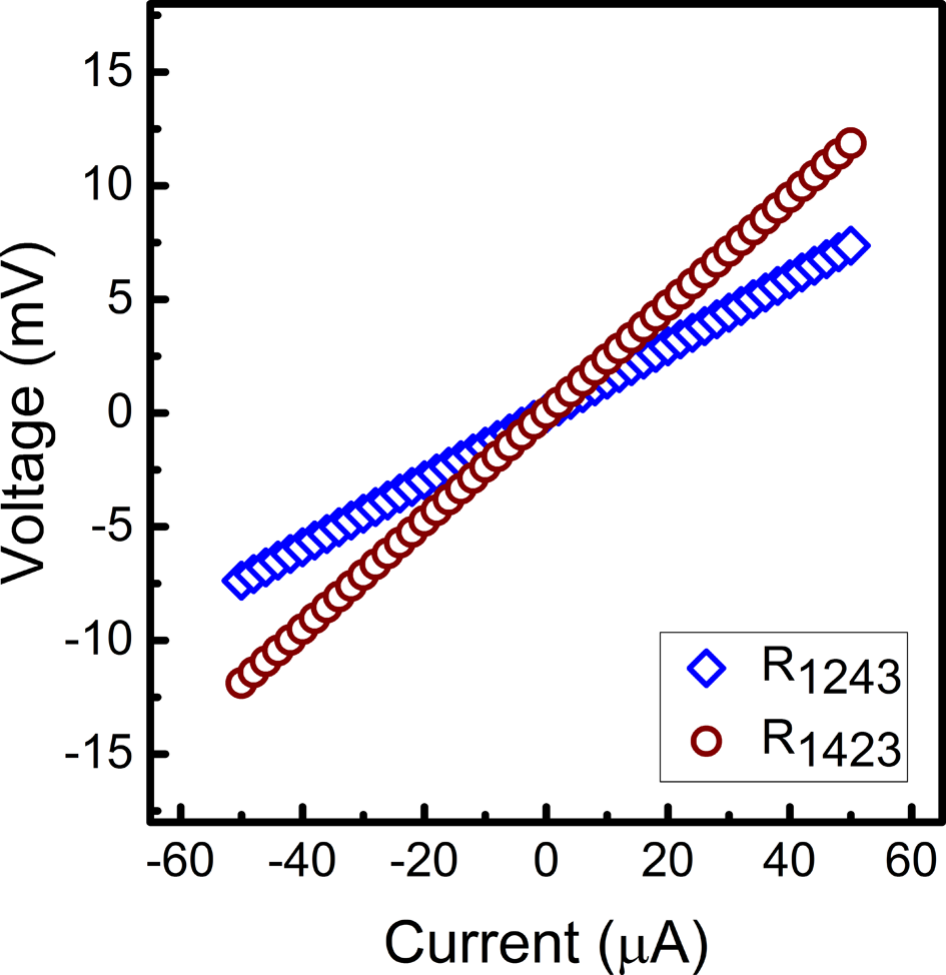
*I*–*V* characteristics of vertical graphene nanosheets from four-probe measurements.

The sheet resistance of VGNs was measured to study their electrical properties. The linear current–voltage relationship from the four-probe resistance measurement confirms the ohmic behavior of all the studied samples (Figure 10). The NG film, grown at 600 °C, is also found to be electrically conducting with a sheet resistance of 5.6 kΩ/sq. The sheet resistance of this film is lower than that of a few-layer graphene (9.1 kΩ/sq for three layers) reported by Peng and co-workers [63]. Such direct growth on an insulating substrate at low temperature without post-growth treatment offers a good compatibility with the semiconductor processing technologies. A sheet resistance of 0.98 kΩ/sq is observed for the VGNs grown at 800 °C. The lowest sheet resistance of 0.6 kΩ/sq is obtained for the film grown at a distance of 10 cm from the plasma source, which is lower than that of CVD-grown planar graphene with eight layers (0.77 kΩ/sq) [64]. Grain boundary and edge defects and disordered carbon, and mainly the sp^3^ content play key roles in determining the resistivity [9]. Here, the high resistivity of the sample grown at 600 °C can be explained by grain boundary and edge defects of NG, which diminish electron mobility. On the other hand, higher growth temperatures improved the crystallinity and resulted in lower resistivity.

## Conclusion

In summary, plasma-enhanced chemical vapor deposition (PECVD) was employed to conduct a series of controlled growth experiments. The experimental results specify the effects of the growth temperature, plasma power and distance from plasma source to substrate on the morphology and structure of vertical graphene nanosheets (VGNs). Systematic microscopic and spectroscopic investigations, wetting and electrical studies ensure that the structure of the self-organized VGNs affects their physical properties. The factors contributing towards this are mainly surface adatom mobility, long-lived species, dissociation rate of gas(es) in the plasma, C/H ratios, and transport of the plasma species. The activation energy for the vertical growth is found to be 0.57 eV. The decreasing trend of residual compressive stress for vertical growth with growth temperature suggests that the intrinsic electric field plays a major role in the pronounced vertical growth compared to the effects of stress released at grain boundaries at higher temperature. It is also shown that the morphology (from planar to vertical networks) wetting, and structural and electrical properties of VGNs can be tuned during the same growth process while retaining the amount of sp^3^ content in the structure. The established correlation provides better understanding on the growth of VGNs with the optimized and controlled morphology of interest to the diverse envisaged applications.

## Experimental

### Synthesis of VGNs

ECR-PECVD was employed for the growth of VGNs on thermally oxidized SiO_2_/Si substrates. The SiO_2_ layer was grown on single-crystal Si substrates by thermal oxidation and the thickness of the oxide layer is maintained at around 400 nm by controlling oxidation time. Ar and CH_4_ of high purity were used as dilution and source gas, respectively. Prior to the growth, the substrates were cleaned by acetone, isopropyl alcohol and deionized water, followed by drying with N_2_ gas, and then loaded into the growth chamber. The chamber was evacuated to 10^−6^ mbar. The substrate was subjected to annealing and subsequently cleaned by Ar plasma with 200 W power at the respective temperatures. CH_4_/Ar ratio, growth time and annealing conditions (before and after growth) were kept constant for this study. The work was classified into three cases, as shown in Table 1. The distance from plasma source to substrates is denoted as the “distance” throughout the manuscript for the sake of simplicity. The surface of the dielectric cup is taken as a reference point at which the plasma discharge occurs. The substrates were annealed after the growth for 30 min at the respective growth temperature by just switching off the microwave plasma. Finally, the substrates were allowed to cool down to room temperature and samples were taken out from the vacuum chamber for further characterization.

**Table 1:** Process parameters for VGNs growth by ECR-PECVD.

parameter	value

CH_4_/Ar gas flow (sccm)	5/25
Gas pressure (mbar)	3 × 10^−3^
Growth time (min)	30
case I: growth temperature *T* (°C) at *d* = 30 cm and *P* = 320 W	600, 625, 650, 725, 800
case II: distance *d* (cm) at constant *P* = 320 W and *T* = 800 °C	10, 20, 30, 40
case III: microwave power *P* (W) at *d* = 30 cm and *T* = 800 °C	200, 280, 320, 375, 425, 475
annealing time (min) at respective growth temperature	30

### Characterization of VGNs

Morphological features of these films were examined by field-emission scanning electron microscopy (Supra 55, Carl Zeiss, Germany). High-resolution transmission electron spectroscopy (Libra 200 FE, Carl Zeiss, Germany) was used to investigate the microstructure of the films.

A micro-Raman spectrometer (Renishaw inVia, UK) was used to evaluate the structural properties in terms of defects and disorder. In backscattering geometry, Raman spectroscopic data were recorded in the frequency region of 1000–3500 cm^−1^ for 30 s accumulation time using a 514 nm laser and 100× objective lens.

The wetting properties of the films were measured by the sessile drop method with the help of a CCD camera (Apex Instrument Co. Pvt. Ltd., India). The volume of the droplet was about 1 µL and all measurements were carried out under ambient conditions. The value of contact angle was evaluated by half angle fitting method prescribed by Apex instrument.

Ag paste was used to make four-probe contacts and an Agilent B2902A precision source/measure unit was used to estimate sheet resistance in van der Pauw geometry.

### Author contributions

S. G. planned, performed the experiments, analyzed the data and wrote the manuscript. S. R. P. carried out scanning electron microscopy. S. R. P., N. K. and K. O. contributed to the data analysis and interpretation and manuscript preparation. All authors discussed the results, commented on the manuscript and gave approval to the final version of the manuscript.

## References

[1] Bo Z, Mao S, Jun Han Z, Cen K, Chen J, Ostrikov K (2015). Chem Soc Rev.

[2] González Z, Vizireanu S, Dinescu G, Blanco C, Santamaría R (2012). Nano Energy.

[3] Notarianni M, Liu J, Vernon K, Motta N (2016). Beilstein J Nanotechnol.

[4] Mishra K K, Ghosh S, Thoguluva R R, Amirthapandian S, Kamruddin M (2016). J Phys Chem C.

[5] Ghosh S, Ganesan K, Polaki S R, Sivadasan A K, Kamruddin M, Tyagi A K (2016). Adv Sci, Eng Med.

[6] Ghosh S, Mathews T, Gupta B, Das A, Krishna N G, Kamruddin M (2017). Nano-Struct Nano-Objects.

[7] Levchenko I, Beilis I I, Keidar M (2016). Adv Mater Technol (Weinheim, Ger).

[8] Soin N, Roy S S, Sharma S, Thundat T, McLaughlin J A (2013). J Solid State Electrochem.

[9] Cho H J, Kondo H, Ishikawa K, Sekine M, Hiramatsu M, Hori M (2014). Carbon.

[10] Krivchenko V A, Evlashin S A, Mironovich K V, Verbitskiy N I, Nefedov A, Wöll C, Kozmenkova A Ya, Suetin N V, Svyakhovskiy S E, Vyalikh D V (2013). Sci Rep.

[11] Seo D H, Kumar S, Rider A E, Han Z, Ostrikov K K (2012). Opt Mater Express.

[12] Shih W-C, Jeng J-M, Huang C-T, Lo J-T (2010). Vacuum.

[13] Watanabe H, Kondo H, Okamoto Y, Hiramatsu M, Sekine M, Baba Y, Hori M (2014). Appl Phys Lett.

[14] Bo Z, Tian Y, Han Z J, Wu S, Zhang S, Yan J, Cen K, Ostrikov K (2017). Nanoscale Horiz.

[15] Bo Z, Yang Y, Chen J, Yu K, Yan J, Cen K (2013). Nanoscale.

[16] Ando Y, Zhao X, Ohkohchi M (1997). Carbon.

[17] Wu Y, Qiao P, Chong T, Shen Z (2002). Adv Mater.

[18] Hiramatsu M, Hori M (2010). Carbon nanowalls: synthesis and emerging applications.

[19] Jacob M V, Rawat R S, Ouyang B, Bazaka K, Kumar D S, Taguchi D, Iwamoto M, Neupane R, Varghese O K (2015). Nano Lett.

[20] Levchenko I, Ostrikov K K, Zheng J, Li X, Keidar M, Teo K B (2016). Nanoscale.

[21] Zhu M, Wang J, Holloway B C, Outlaw R A, Zhao X, Hou K, Shutthanandan V, Manos D M (2007). Carbon.

[22] Malesevic A, Vitchev R, Schouteden K, Volodin A, Zhang L, Van Tendeloo G, Vanhulsel A, Van Haesendonck C (2008). Nanotechnology.

[23] Zhao J, Shaygan M, Eckert J, Meyyappan M, Rümmeli M H (2014). Nano Lett.

[24] Ghosh S, Ganesan K, Polaki S R, Mathews T, Dhara S, Kamruddin M, Tyagi A K (2015). Appl Surf Sci.

[25] Krivchenko V V, Dvorkin V A, Dzbanovsky N N, Timofeyev M A, Stepanov A S, Rakhimov A T, Suetin N V, Vilkov O Yu, Yashina L V (2012). Carbon.

[26] Muñoz R, Gómez-Aleixandre C (2014). J Phys D: Appl Phys.

[27] Ghosh S, Ganesan K, Polaki S R, Ravindran T R, Krishna N G, Kamruddin M, Tyagi A K (2014). J Raman Spectrosc.

[28] Uchida T, Baliyan A, Fukuda T, Nakajima Y, Yoshida Y (2014). RSC Adv.

[29] Xiong G, Hembram K P S S, Zakharov D N, Reifenberger R G, Fisher T S (2012). Diamond Relat Mater.

[30] Yang C, Bi H, Wan D, Huang F, Xie X, Jiang M (2013). J Mater Chem A.

[31] Park H J, Kim T Y, Lee J W, Ahn B U, Jung Y H, Choi Y S, Song Y I, Suh S J (2015). Thin Solid Films.

[32] Sumi H, Ogawa S, Sato M, Saikubo A, Ikenaga E, Nihei M, Takakuwa Y (2010). Jpn J Appl Phys.

[33] Terasawa T-o, Saiki K (2012). Carbon.

[34] Srivastava S K, Shukla A K, Vankar V D, Kumar V (2005). Thin Solid Films.

[35] Kim Y S, Lee J H, Kim Y D, Jerng S-K, Joo K, Kim E, Jung J, Yoon E, Park Y D, Seoab S (2013). Nanoscale.

[36] Vizireanu S, Mitu B, Luculescu C R, Nistor L C, Dinescu G (2012). Surf Coat Technol.

[37] Ubnoske S M, Raut A S, Brown B, Parker C B, Stoner B R, Glass J T (2014). J Phys Chem C.

[38] Wang B B, Zheng K, Cheng Q J, Ostrikov K (2015). Appl Surf Sci.

[39] Vizireanu S, Stoica S D, Luculescu C, Nistor L C, Mitu B, Dinescu G (2010). Plasma Sources Sci Technol.

[40] Vizireanu S, Stoica S D, Mitu B, Husanu M A, Galca A, Nistor L, Dinescu G (2009). Appl Surf Sci.

[41] Banerjee A, Das D (2013). Appl Surf Sci.

[42] Kim S Y, Choi W S, Lee J-H, Hong B (2014). Mater Res Bull.

[43] Wang J, Zhu M, Outlaw R A, Zhao X, Manos D M, Holloway B C (2004). Carbon.

[44] Tian M, Batty S, Shang C (2013). Carbon.

[45] Mironovich K V, Itkis D M, Semenenko D A, Dagesian S A, Yashina L V, Kataev E Yu, Mankelevich Y A, Suetin N V, Krivchenko V A (2014). Phys Chem Chem Phys.

[46] Liu D, Yang W, Zhang L, Zhang J, Meng J, Yang R, Zhang G, Shi D (2014). Carbon.

[47] Ghosh S, Ganesan K, Polaki S R, Ilango S, Amirthapandian S, Dhara S, Kamruddin M, Tyagi A K (2015). RSC Adv.

[48] Wang F-J, Deng L-N, Deng J-H (2015). Appl Surf Sci.

[49] Cai M, Outlaw R A, Quinlan R A, Premathilake D, Butler S M, Miller J R (2014). ACS Nano.

[50] Thomas R, Rao G M (2015). RSC Adv.

[51] Chen J, Bo Z, Lu G (2015). Vertically-Oriented Graphene.

[52] Hung T-C, Chen C-F, Chen C-C, Whang W-T (2009). Appl Surf Sci.

[53] Cançado L G, Jorio A, Pimenta M A (2007). Phys Rev B.

[54] Kariminejad A, Taheri-Nassaj E, Ghanbarian M, Hassanzadeh-Tabrizi S A (2016). Mater Des.

[55] Watanabe H, Kondo H, Hiramatsu M, Sekine M, Kumar S, Ostrikov K, Hori M (2013). Plasma Processes Polym.

[56] Jiang N, Wang H X, Zhang H, Sasaoka H, Nishimura K (2010). J Mater Chem.

[57] Neyts E C (2016). Plasma Chem Plasma Process.

[58] Ostrikov K K, Yoon H-J, Rider A E, Vladimirov S V (2007). Plasma Processes Polym.

[59] Ostrikov K, Neyts E C, Meyyappan M (2013). Adv Phys.

[60] Levchenko I, Ostrikov K, Keidar M, Xu S (2005). J Appl Phys.

[61] Liu W, Dang T, Xiao Z, Li X, Zhu C, Wang X (2011). Carbon.

[62] Zhang L X, Sun Z, Qi J L, Shi J M, Hao T D, Feng J C (2016). Carbon.

[63] Peng K-J, Wu C-L, Lin Y-H, Liu Y-J, Tsai D-P, Pai Y-H, Lin G-R (2013). J Mater Chem C.

[64] Reina A, Jia X, Ho J, Nezich D, Son H, Bulovic V, Dresselhaus M S, Kong J (2009). Nano Lett.

